# Non-ketotic Hyperglycemic Hemichorea as a Clue to Undiagnosed Type 2 Diabetes Mellitus in an Elderly Man: A Case Report

**DOI:** 10.7759/cureus.93391

**Published:** 2025-09-28

**Authors:** Rafael Machado, Rita Marçal

**Affiliations:** 1 Family Medicine, Centro de Saúde de Câmara de Lobos, Serviço de Saúde da Região Autónoma da Madeira, Câmara de Lobos, PRT; 2 Family Medicine, Centro de Saúde do Caniçal, Serviço de Saúde da Região Autónoma da Madeira, Caniçal, PRT

**Keywords:** case report, diabetic striatopathy, hemichorea-hemiballismus, non-ketotic hyperglycemic hemichorea, primary care follow-up, type 2 diabetes mellitus (dm)

## Abstract

Non‑ketotic hyperglycemic hemichorea is a rare neurologic complication associated with poorly controlled and, in exceptional cases, previously undiagnosed type 2 diabetes, characterized by sudden unilateral choreiform movements and basal ganglia hyperdensity on CT or T1-hyperintensity on MRI. We report the case of a 76‑year‑old male without routine primary-care follow-up who presented to the emergency department after one week of left-arm choreo‑dystonic movements, accompanied by mild left-sided hemiparesis and hypoesthesia. Laboratory evaluation revealed severe hyperglycemia (381 mg/dl) and glycosuria but no ketonuria. Non-contrast brain CT identified a discrete hyperdensity in the right caudate and lentiform nucleus, with no additional findings to suggest hemorrhagic or ischemic stroke. A 6-unit intravenous bolus of regular human insulin normalized serum glucose and markedly reduced neurological symptoms. These findings were consistent with non‑ketotic hyperglycemic hemichorea. Following complete resolution of hemichorea at discharge, the patient underwent glycemic optimization with insulin and oral antidiabetic agents, achieving HbA1c normalization within seven months and transitioning to primary care follow-up without recurrence of neurological symptoms. This case highlights hemichorea as a possible initial manifestation of undiagnosed diabetes, especially in an elderly man, and underscores the need for systematic metabolic screening in primary-care settings to facilitate early diagnosis and prevent such atypical presentations.

## Introduction

Hemichorea is a hyperkinetic movement syndrome characterized by abrupt, involuntary, non-patterned, and purposeless movements affecting one side of the body [1,2]. This condition results from dysfunction of the contralateral basal ganglia, often caused by underlying structural lesions such as vascular insults, neoplasms, drug effects, autoimmune diseases, or metabolic disturbances [1,2]. 

Non-ketotic hyperglycemic hemichorea (NKH-HC) is a rare neurological complication of longstanding, poorly controlled, and, in exceptional cases, previously undiagnosed type 2 diabetes mellitus [3], with a reported prevalence of approximately one case per 100,000 individuals [4], affecting most commonly elderly women [2,5]. 

Clinically, NKH-HC is marked by an acute onset of unilateral involuntary choreiform movements of the limbs. Neuroimaging typically reveals distinctive findings in the contralateral basal ganglia, such as hyperdensity on CT or hyperintense signals on T1-weighted MRI, reflecting perfusion changes in these regions that have recently been termed “diabetic striatopathy” [1,6]. The most accepted pathogenic hypothesis suggests a shift toward anaerobic cerebral metabolism, leading to rapid depletion of gamma‑aminobutyric acid (GABA) and subsequent disinhibition of the subthalamic nucleus and basal ganglia. In non-ketotic states, the lack of ketone bodies prevents GABA resynthesis, worsening this imbalance [2,4,5]. Other pathological findings, such as microvascular hemorrhage and reactive astrocytosis, further suggest that vascular injury and gliosis may also contribute to the clinical and imaging features of the condition [2,4,5]. 

Recognizing these clinical and radiological hallmarks is essential because prompt correction of hyperglycemia typically leads to a rapid, often complete, symptom resolution, although recurrence has been reported in approximately 18 % of cases [4]. 

## Case presentation

A 76-year-old man presented to the emergency department with continuous, abnormal, involuntary movements of the left arm that had persisted for one week. He denied headaches, dizziness, loss of consciousness, or dysarthria. Past medical history included hypertension and surgical drainage of a left traumatic subdural hematoma four years earlier, with no medical follow-up thereafter. He reported no regular primary care visits for the past five years, with medication non-adherence during that period. He also reported no recent head trauma.

On examination, the patient was awake, alert, and fully oriented. Vital signs were within normal limits. Neurologic assessment revealed non-stereotyped choreic-dystonic movements of the left upper and lower limbs, accompanied by mild left-sided hemiparesis and hypoesthesia. 

Initial blood and urinary tests revealed a serum glucose level of 381 mg/dL, glycosuria, and negative urinary ketone bodies. Complete blood count, corrected sodium, arterial blood gas test, electrolytes, and renal and hepatic panels were all within normal limits. Thyroid tests and HbA1c were not available in the emergency setting.

**Figure 1:**
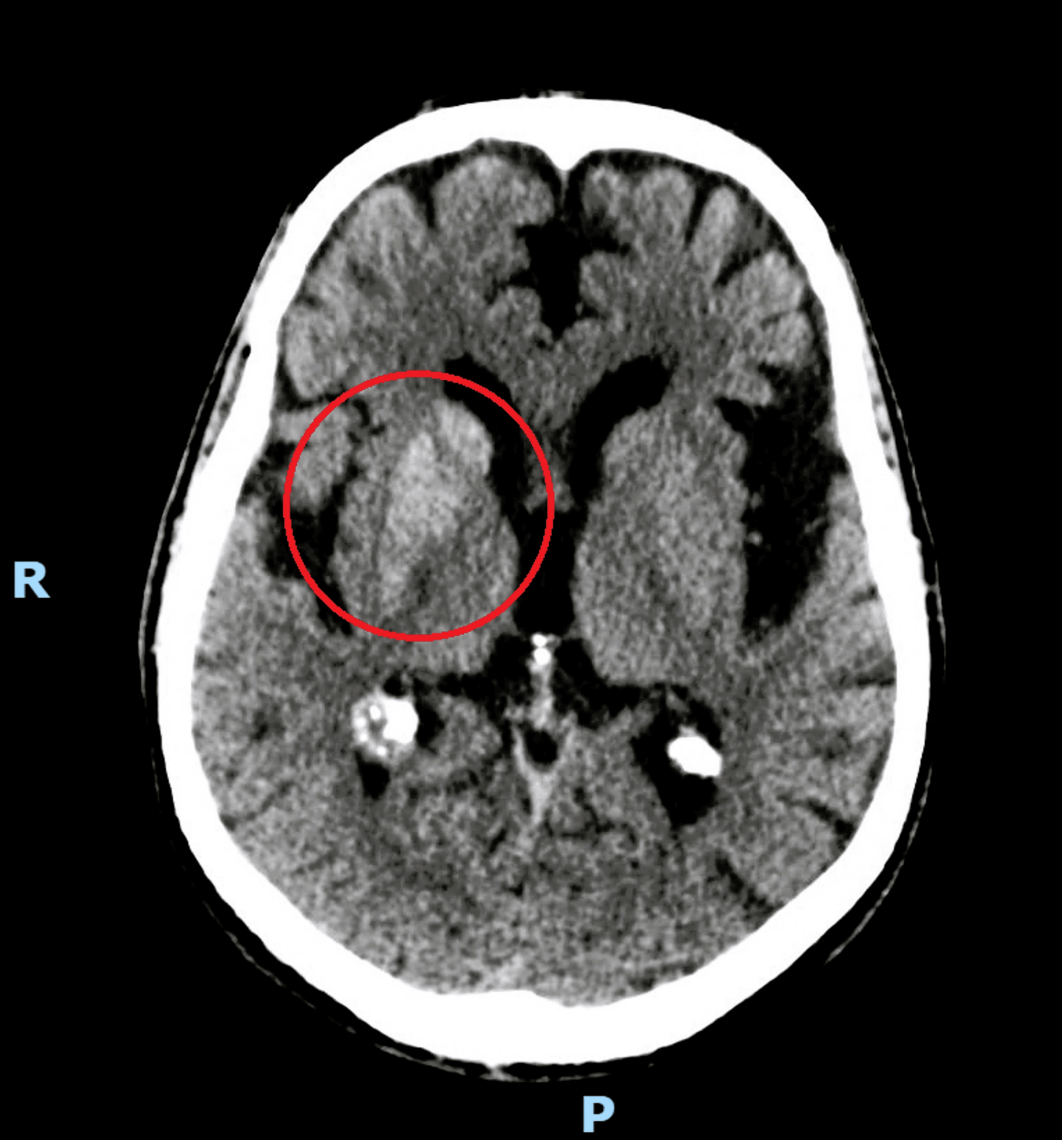
Single axial non-contrast head CT shows a discrete hyperdensity involving the right caudate and lentiform nucleus, circled in red This non-contrast head CT scan was performed with 3 mm slices parallel to the orbitomeatal plane.

Non-contrast head CT showed a discrete focal hyperdensity involving the right caudate and lentiform nucleus (Figure 1), together with incipient signs of microangiopathic subcortical leukoencephalopathy and postoperative sequelae from the prior left frontal-parietal trepanation. There was no evidence of mass effect, midline shift, or extra-axial collections. The ventriculocisternal system was patent. MRI was not available in the emergency department. These findings were consistent with a probable diagnosis of non-ketotic hyperglycemic hemichorea. 

The patient received a 6-unit bolus of human insulin (100 U/mL) followed by an isotonic saline infusion, guided by hydration status. Glucose levels were monitored every two hours. Six hours later, his serum glucose had reduced to 196 mg/dL, and the intensity of his neurological symptoms had markedly diminished. By the time of discharge from the emergency department, hemichorea had resolved completely. The patient was prescribed metformin 500 mg twice daily, and he was referred for outpatient endocrinology follow‑up.

Three days after discharge, at the endocrinology appointment, HbA1c was 14.2%, fasting plasma glucose 381 mg/dL, and thyroid panel within normal range. Insulin glargine was started at 18 units nightly. After one month, HbA1c decreased to 11.8%, insulin was maintained, and metformin was replaced with a fixed-dose combination of metformin/sitagliptin 850/50 mg twice daily. At seven months, HbA1c had normalized to 5.6% and no hypoglycemic episodes were reported. Insulin was discontinued, and the oral antidiabetic was increased to 1000/50 mg twice daily. The patient was discharged from the endocrinology care and enrolled for ongoing surveillance at his primary‑care health center. There was no report of neurologic signs.

## Discussion

This case highlights NKH‑HC as an uncommon initial manifestation of undiagnosed diabetes, particularly in an elderly individual lacking regular medical care. The presentation in this patient is atypical, as most reported cases occur in elderly women with long-standing type 2 diabetes mellitus [2,5]. However, recent meta-analyses and case series suggest that the gender distribution may be more balanced than previously thought, and that NKH-HC can also be the first clinical sign of diabetes in previously undiagnosed individuals [4].

The main differential diagnoses for hemichorea include acute ischemic stroke, intracerebral hemorrhage, basal ganglia tumors, or metastases. In this patient, the characteristic CT hyperdensity localized to the basal ganglia, without mass effect, midline shift, or extra-axial collections, correlated with marked hyperglycemia, strongly supports NKH‑HC. Unlike structural lesions such as neoplastic or hemorrhagic processes, this imaging pattern is consistent with previous reports, which lack mass effect and often spares the internal capsule [2,7].

Most patients experience rapid symptom resolution following normalization of blood glucose levels, as seen in this case. However, recurrence is not uncommon, with rates reported up to 18% [4]. Factors contributing to recurrence may include poor glycemic control, delayed initial treatment, and underlying striatal atrophy in imaging, which may predispose the basal ganglia to dysfunction [7].

Treatment typically involves glycemic control, often with insulin, and recovery time was significantly shorter in patients treated with glucose control alone, suggesting that early intervention may prevent progression to more severe or persistent symptoms [4].

From a primary care perspective, this case underscores the importance of opportunistic diabetes screening, especially in elderly patients with limited healthcare access. Early detection and management of diabetes can substantially reduce complications such as NKH-HC and reduce the burden of disease. Strengthening primary care infrastructure, therefore, remains pivotal for routine monitoring, patient education, and early identification of medication non‑adherence or disease decompensation, ultimately improving long-term outcomes. 

## Conclusions

Hemichorea should prompt clinicians to assess hyperglycemia, especially in elderly patients without an established diagnosis of diabetes. Contralateral basal ganglia hyperdensity on CT together with hyperglycemia suggests NKH-HC. Timely glycemic correction usually resolves the movement disorder. This case also emphasizes the critical role of continuous healthcare engagement, as systematic diabetes screening in primary care is essential for early diagnosis and complication prevention. 
